# One-time-pad cryptography scheme based on a three-dimensional DNA self-assembly pyramid structure

**DOI:** 10.1371/journal.pone.0206612

**Published:** 2018-11-06

**Authors:** Weiping Peng, Danhua Cheng, Cheng Song

**Affiliations:** School of Computer Science and Technology, Henan Polytechnic University, Jiaozuo, Henan, China; King Saud University, SAUDI ARABIA

## Abstract

The security strength of the traditional one-time-pad encryption system depends on the randomness of the secret key. However, It can hardly to generatea truerandom key by using the existing technologies and methods, and it is also difficult to issue and store the random keywhich is at least as long as the plaintext. Therefore, we pay more attention to the logical operation used in the encryption and decryption but not to how to generate the random key. The calculator, a three-dimensional DNA self-assembly pyramid structure, is designed to construct four common logical operations (AND, OR, NOT, XOR) by programming DNA interactions. And two novel one-time-pad cryptography schemes, a single-bit one-time-pad algorithm and improved double-bit one-time-pad algorithm, are proposed based on the calculator. The security fragments, used to construct the three-dimensional DNA self-assembly pyramid structure, are intercepted from a reference chain which is selected from the DNA database. All of the interception parameters are transmitted to recipient by hiding in DNA sequences. Only the authorized user can get all secret parameters to reconstruct the structure. The secret random key sequences for the two one-time-pad cryptography algorithms are generated by using logistic map. It only needs to share two parameters and thresholding function in sender and recipient without code books. The simulation results and security analysis show that the encryption algorithms are effective and can provide higher computational complexity as well as a reduced cracking probability except for the difficult of biological experiments.

## Introduction

DNA cryptography, accompanied by the DNA computing [1–3], is an emerging direction in the field of information security [4]. The vast parallelism [1] and extraordinary information density inherent in DNA molecules are explored for cryptographic purposes such as encryption, authentication, and signature and so on [2]. Comparing with the traditional cryptography and quantum cryptography, the significant difference is that the DNA cryptography provides higher security and feasibility mainly depends on the biological difficult problems. Many DNA cryptography algorithms, combined with the molecular calculation of DNA [5–10] and the traditional cryptographic algorithm, have been designed and realized since DNA as molecular programming reagent was used to resolve the seven-city Hamilton path problem [2]. All of the proposed one-time-pad DNA cryptosystems can be divided into two types: one is only make DNA database as a codebook to build a one-time-pad DNA cryptosystem and the other is to construct a brand new DNA cryptosystem based on some biological difficult problems [11]. The logical operations of one-time-pad DNA cryptosystems include substitution method [12] and XOR operation method [12, 13]. The one-time-pad cryptosystem based on the substitution method uses the DNA database as a codebook to construct a mapping table which possesses a unique characteristic of the inverse mapping. The plaintext will be divided into a fixed length by using the mapping mechanism and replaced with the corresponding DNA ciphertext. The ciphertext can be decrypted according to the inverse mapping. Each mapping table represents a mapping between a segment of plaintext and ciphertext.

The one-time-pad cryptosystem based on the XOR operation [14] makes use of many DNA sequences as one-time-pad codebook. There DNA sequences are composed of a number of evenly distributed random bit sequence. The XOR logic operation is constructed by using the biological properties of DNA base and strand displacement techniques (using the principle ideas of central dogma of molecular biology [9] and theoretical [9, 12] or biological experiments [13]). For example, the cryptography method [9] did not use real DNA computing but just used the principle of central dogma of molecular biology. Gehani [12] designed two types of one-time-pad DNA encryption technologies by using mapping substitution and Exclusive-OR method respectively. Jing Yang et al [13] designed an XOR logical gate based on a DNA self-assembly structure by using DNA strand displacement and then proposed a one-time-pad scheme. In addition to designing a secure and efficient mapping and XOR operation scheme, the two methods mentioned above need to construct a large amount of one-time-pad codebook with strong randomness. Rao Nini [15] designed a cryptosystem based on recombinant DNA technique, and the process of encryption and decryption was completed in the DNA recombination. Wang et al [16] designed a one-time-pad algorithm based on a codebook which synthesized several DNA strands of length *N* (*N*>40).

In this work we proposed two novel one-time-pad DNA cryptography schemes based on a DNA calculator. The calculator, a three-dimensional DNA self-assembly pyramid structure, is designed to construct four common logical operations (AND, OR, NOT, XOR) by programming DNA interactions. These single-stranded DNA (ssDNA) molecules are used as input signals, and the release of a ssDNA molecule after a series of toehold directed strand displacement reactions as a detectable output signal. Compared with some similar encryption schemes in the existing literature, the main difference is that the three-dimensional DNA self-assembly structure is more complex but simple to implement. The security fragments, used to construct the DNA self-assembly pyramid structure, were intercepted from a reference chain which was selected from the DNA database. All of the interception parameters were transmitted to recipient by hiding in a DNA sequence. Only the authorized user can get all secret parameters to reconstruct the structure. The secret random key sequences for the two one-time-pad cryptography algorithms were generated by using logistic map. It just only needs to share two parameters and thresholding function in encryption and decryption without code books. Furthermore, combined with the three factor (3FA) remote user authentication protocol proposed by Siddiqui Z et al. [17], we can establish a virtual DNA experimental platform on the private cloud to provide DNA self-assembly structure and strand displacement reaction, and design a four factor (4FA) remote authentication protocol by using the user-specific fluorescence spectrum as the fourth factor of authentication based on the experimental platform. The 4FA authentication scheme can provide dual security in both computational and biological. The example results and security analysis show that the encryption algorithms are effective and can provide higher computational complexity as well as a reduced cracking probability except for the difficult of biological experiments.

This paper mainly explains the encryption and decryption scheme. The remaining part of this paper is organized as follows. Section 2 describes a construction method of three-dimensional DNA self-assembly pyramid structure. Then, four different logical operations are constructed by the strand displacement based on the self-assembly structure. In Sections 3 and 4, two one-time-pad cryptography schemes based on single-bit and double-bit are designed. The encryption and decryption processes are described in detail below. Section 5 analyzes the security and characteristics. The conclusions of this work are presented in Section 6.

## The construction of calculator

**Fig 1:**
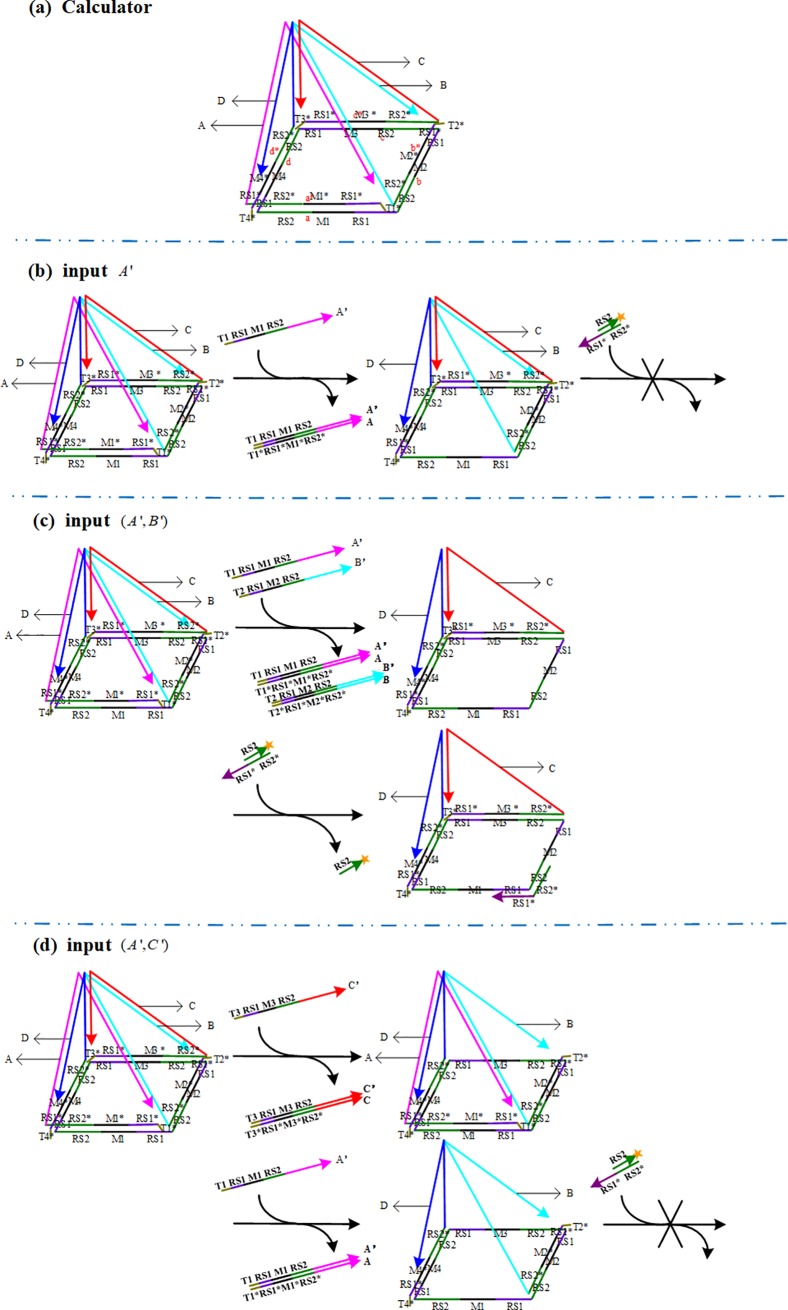
Architectural design of the calculator based on DNA strand displacement reactions. (a) The pentahedral pyramid DNA self-assembly structure. (b) The displacement process of a single input strand *A*'. (c) The displacement process of the adjacent strands (*A*',*B*'). (d) The displacement process of the relative strands (*A*',*C*').

Referring to the reference [6] and [7], a pentahedral pyramid DNA self-assembly structure is constructed (see Fig 1(A)). The pentahedral self-assembly structure consists of a bottom square and four lateral triangles. The bottom circular ssDNA are composed of four edges marked as *a*(*RS*_1_-*M*_1_-*RS*_2_), *b*(*RS*_1_-*M*_2_-*RS*_2_), *c*(*RS*_1_-*M*_3_-*RS*_2_), and *d*(*RS*_1_-*M*_4_-*RS*_2_). The length of each edge is 42nts. *RS*_1_ and *RS*_2_ are 8*nts* and 18*nts*, respectively.

The *RS*_1_-*RS*_2_ joint sequence is repeated four times in the self-assembly structure. [*M*_1_,*M*_2_,*M*_3_,*M*_4_] are each 16*nts* long and have distinct sequences. The structure can be changed randomly according to the different parameters of [*M*_1_,*M*_2_,*M*_3_,*M*_4_]. Each lateral triangle of the structure is a completed sequence and marked as [*A*,*B*,*C*,*D*]. The bases of four lateral triangles are named [a*,b*,c*,d*] which are perfectly complementary to [*a*,*b*,*c*,*d*], respectively. Each base consists of two parts: the part one, *RS*_1_*-*M*_i_*-*RS*_2_*(*i* = 1,2,3,4), is fully complementary to [*a*,*b*,*c*,*d*]; the other, [*T*_1_*,*T*_2_*,*T*_3_*,*T*_4_*], is a unique sequence toehold for initiating the computation process. The other two sides of the triangle are perfectly complementary to the sides of the adjacent triangle. The ssDNA *RS*_1_-*RS*_2_ and *T*_i_* are invariable and reusable. It only needs to change the sequences *M*_*i*_(*i* = 1,2,3,4) so as to construct different structures.

According to the characteristics and principles of DNA strand displacement, strand displacement is realized when the complementary strand of the relevant chain is added into the medium containing the self-assembly structure. By adding the special fluorescence detector which is a duplex of *RS*_2_ and *RS*_1_*-*RS*_2_*, we can distinguish the output of the results by observing whether an obvious fluorescence signal is present.

The design principle of the calculator is that the fluorescence signal can be detected in the reaction solution by adding "detector" [6] if one of the *RS*_1_-*RS*_2_ joints is exposed. Otherwise, the fluorescent signal cannot be obtained.

We note that [*A*',*B*',*C*',*D*'] are fully complementary to [*A*,*B*,*C*,*D*], respectively. Without loss of generality, if [*A*',*B*',*C*',*D*'] are treated as input, there are three different situations according to the number of chains and the relationship between two chains.

Scene 1: Add only one type of single-strand to the medium of the self-assembly structure.

For example, only strand *A*' is added as input. The toehold *T*_1_ hybridizes with *T*_1_*, whereas only *A* is released by input *A*'. Thus, no *RS*_1_-*RS*_2_ joint is exposed. There will be no significant fluorescence signal after "detector" is added (Fig 1(B)).

Scene 2: Add two different types of single-strands randomly to the medium of the self-assembly structure.

According to the pentahedral pyramid structure, there are two types of positional relationships between two single-strands: adjacent and relative. The adjacent strands include (*A*',*B*'), (*B*',*C*'), (*C*',*D*') and (*A*',*D*'). For example, (*A*',*B*') are used as the adjacent strands and (*A*',*C*') as the relative strands.

If (*A*',*B*') are added, input *A*'and input *B*' are fully complementary to *A* and *B*, respectively. The toeholds, *T*_1_ and *T*_2_, hybridize with *T*_1_* and *T*_2_*. The input strands will fully displace *A* and *B* from the structure. Then, the joint *RS*_1_-*RS*_2_ on the strand will be fully exposed. Significant fluorescence signal will be noted after "detector" is added (Fig 1(C)).

If (*A*',*C*') are added, the chain *A*' and *C*' are fully complementary to *A* and *C*, respectively. Although the input strands fully displace *A* and *C* from the structure, the *RS*_1_-*RS*_2_ joint on the strand cannot be exposed. No significant fluorescence signal is detected after "detector" is added (Fig 1(D)).

Scene 3: Add three or all types of single-strands to the medium of the self-assembly structure.

Obviously, there exist two different single-chains with neighbor relationships if three or more different types of single-strands are randomly selected. According to Fig 1(C), the *RS*_1_-*RS*_2_ joint will appear in the results. After adding the fluorescence detector, there is a significant change in fluorescence intensity.

**Definition 1** Based on the output fluorescence signal after adding the fluorescence detector, we define two output values. The output value is "1" if there is a significant change in the fluorescence intensity; otherwise, the output value is "0".

**Fig 2:**
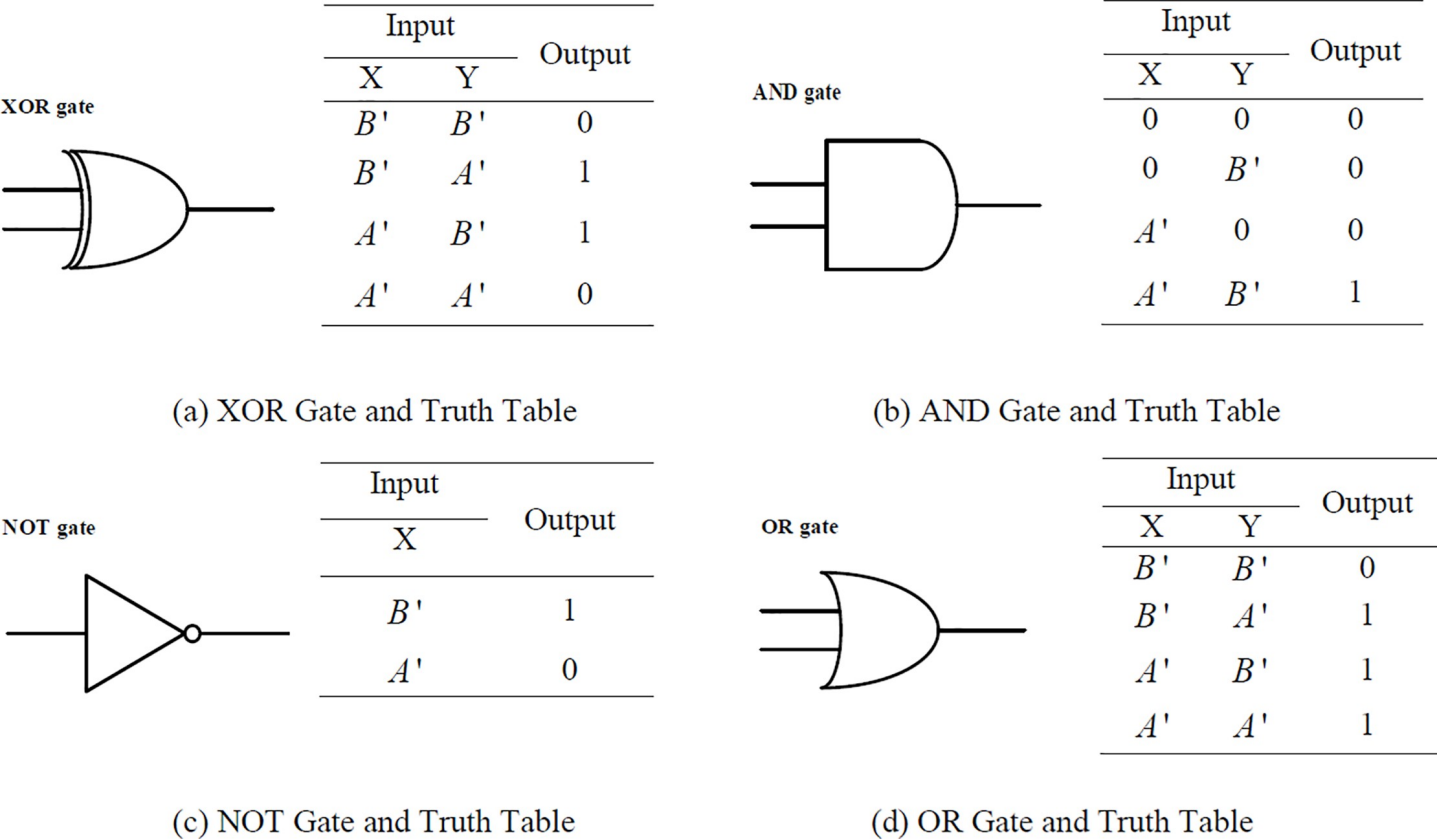
Four logic gates and truth table. (a) Input *A*' and input *B*' are set as "1" and "0", respectively. The output is "1" only upon the simultaneous addition of strands *A*' and *B*'; Otherwise, the output is "0". (b) Both of the input *A*' and input *B*' indicate "1"; "0" indicates no input. (c) Add strand *C*' to displace *C* firstly, and then define input *A*' as "1" and input *B*' as "0". (d) Add strand *D*' to displace *D* first, and then define input *A*' as "1" and input *B*' as "0".

We construct four common logical gate operations (see Fig 2). In the XOR gate (Fig 2(A)), the input *A*' and input *B*' are set as "1" and "0", respectively. The output is "1" only upon the simultaneous addition of stands *A*' and *B*'. Otherwise, the output is "0". In AND gate (Fig 2(B)), both of input *A*' and input *B*' indicate "1"; "0" indicates no input. In NOT gate (Fig 2(C)), add strand *C*' to displace strand *C* firstly, and then input *A*' represents "1" and input *B*' represents "0". In OR gate (Fig 2(D)), add strand *D*' to displace *D* firstly and then define input *A*' as "1" and input *B*'as "0".

## Single-bit one-time-pad encryption model

### Parameter definition

**Table 1** is the definition of the parameters used in the algorithm.

**Table 1:** Parameter definition.

Description	Notation
The stand set and available strand set of the DNA database	*N*, *n*(*n∈N*)
The number of available stands	*λ*
The selected reference strand ID and length	[*p*, *L*]
Four random numbers	[*r*_1_, *r*_2_, *r*_3_, *r*_4_]
Logistic map parameters	[*g*, *μ*]
Four binary sequences	[*x*, *y*, *z*, *u*]
Four DNA sequences	[*M*_*1*_, *M*_*2*_, *M*_*3*_, *M*_*4*_]
Encoding rules	*R*
Primer information	*q*
Encryption and decryption key	*k*
Four input DNA sequences	[*A’*, *B’*, *A"*, *B"*]
Public parameters	*RS*_1_-*RS*_2_ [*T*_1_*,*T*_2_*,*T*_3_*,*T*_4_*]

### Encoding rules

DNA is a long chain polymer consisting of four deoxynucleotides, namely A (adenine deoxynucleotide), T (thymidine deoxynucleotide), C (cytosine deoxynucleotide), and G (guanine deoxynucleotide). The binary code 0 and 1 are complementary, so the digital coding [00,01,10,11] can be used to represent the four bases in the DNA sequence. Obviously, there are 24 combinations. Taking into account the complementary bases *A*-*T* and *C*-*G*, we screen the coding combinations available in **Table 2** below and use one of them at random.

**Table 2:** DNA coding combinations.

code1	code2	code3	code4	code5	code 6	code 7	code 8
00-A	00-A	00-C	00-C	00-G	00-G	00-T	00-T
01-C	01-G	01-A	01-T	01-A	01-T	01-C	01-G
10-G	10-C	10-T	10-A	10-T	10-A	10-G	10-C
11-T	11-T	11-G	11-G	11-C	11-C	11-A	11-A

The plaintext information will be converted into a decimal number according to ASCII table firstly. Then, the decimal number is transformed into a binary string. Finally, the binary string is transformed into the corresponding DNA sequence according to the above DNA digital coding rules.

## Self-assembly structure construction and safe migration

The construction of the self-assembly structure focuses on how to select all of the DNA segments *RS*_1_-*RS*_2_, [*M*_1_,*M*_2_,*M*_3_,*M*_4_], [*A*,*B*,*C*,*D*], and $\left[T_{1}^{*},T_{2}^{*},T_{3}^{*},T_{4}^{*}\right]$. *RS*_1_-*RS*_2_ and $\left[T_{1}^{*},T_{2}^{*},T_{3}^{*},T_{4}^{*}\right]$ can be selected by the reference [5] as open fixed sequences, whereas [*M*_1_,*M*_2_,*M*_3_,*M*_4_] represent secret information and intercept from a reference strand in the DNA database.

(1) Self-assembly structure construction

The steps of the self-assembly structure construction are as follows:

Step 1: According to the selection conditions, all of the available DNA strands are selected from the DNA database to make a set *n*(*n*∈*N*) numbered from 1 to *λ*.

Step 2: Generating a pseudo-random number *x*(1≤*x*≤*λ*). Then, the strand corresponding to *x* is the selected DNA reference strand and recorded as the ID number *p*.

Step 3: Calculating the length *L* of *p*, generating four different pseudo-random numbers *r*_*i*_(*i* = 1,2,3,4) in the interval [1,*L*-16], and intercepting four 16*nts* long DNA segments followed by the starting point *r*_*i*_(*i* = 1,2,3,4) from *p*, named [*M*_1_,*M*_2_,*M*_3_,*M*_4_].

Step 4: Combining [*M*_1_,*M*_2_,*M*_3_,*M*_4_] with the open sequence *RS*_1_-*RS*_2_, the bottom square of the self-assembly structure is constructed.

Step 5: According to the four sides of the bottom square, the four complementary sequences [a*b*c*d*] are obtained as the bottom sides of the side triangles. The other two sides of each triangle only need to be fully complementary to the sides of adjacent triangle.

Through the selection of the above fragments, the self-assembly structure can be completed.

(2) Safe migration of the self-assembly structure

The safe migration of the self-assembly structure is the key to ensuring the confidentiality of the information and the decryption. The authorized user can reconstruct the structure and use the self-assembly structure to complete the decryption operation only when the key set is obtained.

Suppose the recipient possesses the secret parameters (*g*,*μ*,*R*,*p*,*q*) and all the public information. However, the four secret random numbers [*r*_1_,*r*_2_,*r*_3_,*r*_4_] must be obtained first to reconstruct the self-assembly structure. To achieve confidentiality protection, the four random numbers [*r*_1_,*r*_2_,*r*_3_,*r*_4_] are hided in the DNA sequence for transmission with data hiding methods.

The hidden process of random numbers is as follows:

Step 1: Obtaining coding rules *R* and primer information *q* from the secret parameters.

Step 2: Converting the random numbers [*r*_1_,*r*_2_,*r*_3_,*r*_4_] into binary code strings [*x*,*y*,*z*,*u*].

Step3: The binary strings [*x*,*y*,*z*,*u*] are replaced with the corresponding DNA sequences [*s*_1_,*s*_2_,*s*_3_,*s*_4_] according to the mapping of *R*([*A*,*T*,*C*,*G*] and [00,01,10,11]).

Step 4: Connecting the primer information with [*s*_1_,*s*_2_,*s*_3_,*s*_4_] and adding the redundant sequence *s*_5_. Obtaining the DNA sequences {*q*[*s*_1_,*s*_2_,*s*_3_,*s*_4_]*q*'*s*_5_} and transmitting through the public channel.

The extraction process of random numbers is as follows:

Step 1: The authorized user obtains the encoding rules *R* and the primer information *q* through the key set (*K*,*R*,*p*,*q*).

Step 2: Finding the DNA sequences {*q*[*s*_1_,*s*_2_,*s*_3_,*s*_4_]*q*'*s*_5_} in the ciphertext medium according to *q*.

Step 3: Removing the redundancy *s*_5_ from the identified DNA sequences and then converting them into binary strings [*x*,*y*,*z*,*u*] according to *R*.

Step 4: Converting [*x*,*y*,*z*,*u*] to decimal [*r*_1_,*r*_2_,*r*_3_,*r*_4_].

## Single-bit encryption and decryption scheme

**Fig 3:**
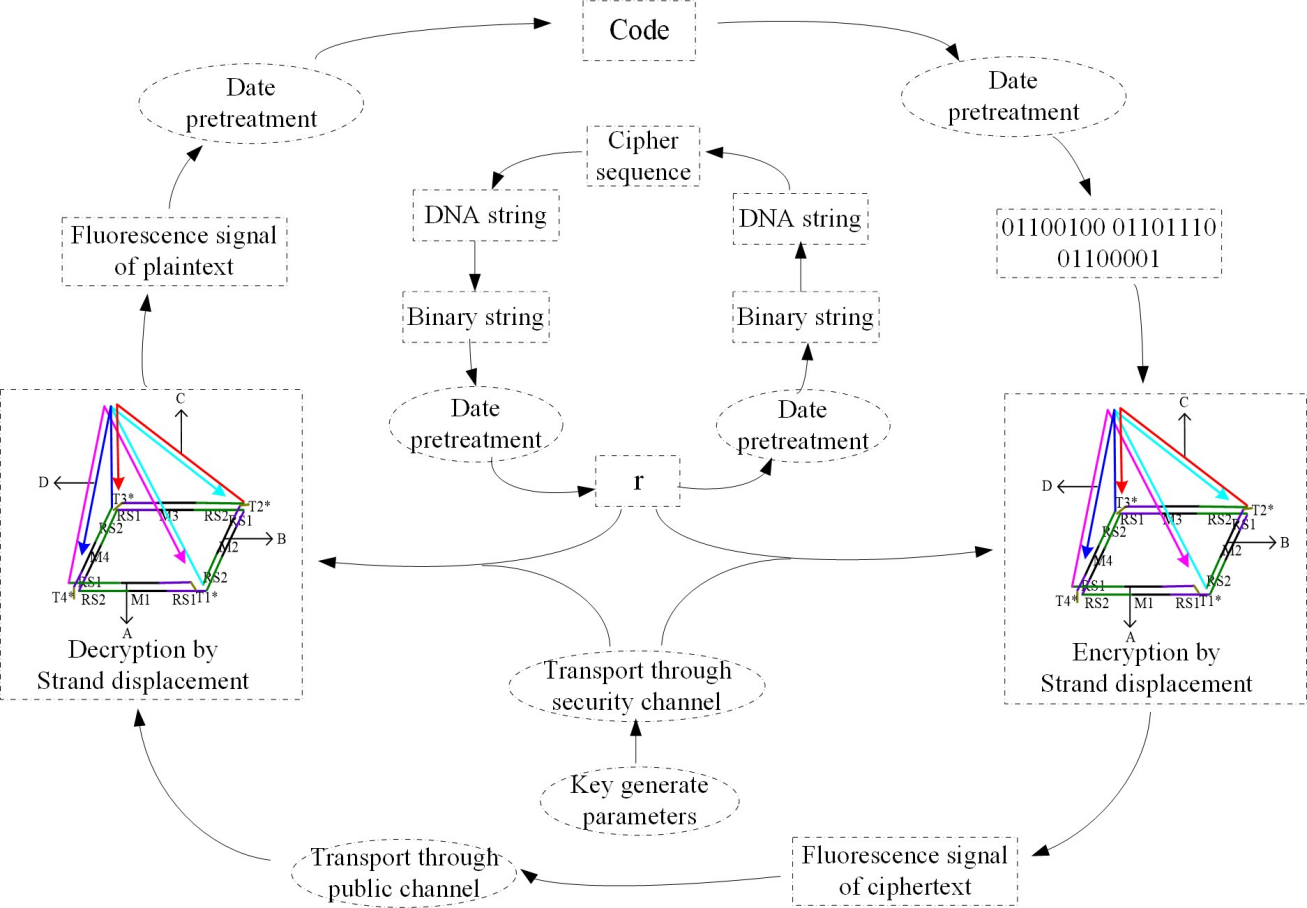
Workflow of DNA cryptography (Encryption process: The plaintext was encrypted with the key after the data processing by using the self-assembly structure; then transmits the fluorescence signal in common channel, and the random numbers were encrypted with steganography and transmitted with the secret information. Decryption process: the decipher first decrypts the random numbers to restructure the self-assembly structure; the key is generated according to the key parameters, then the key and ciphertext are decrypted by XOR operation using the self-assembly structure; the plaintext information is restored after data processing).

Based on the XOR gate rules provided by the above self-assembly structure, the following single-bit one-time-pad cryptography scheme can be constructed. The specific steps of the scheme are illustrated as follows (Fig 3).

(1) System initialization

Suppose Alice is a sender who generates all of the public parameters and the secret parameters. She selects [*M*_1_,*M*_2_,*M*_3_,*M*_4_] from *p* by the random numbers [*r*_1_,*r*_2_,*r*_3_,*r*_4_] and constructs the self-assembly structure with *RS*_1_-*RS*_2_, [*A*,*B*,*C*,*D*] and $\left[T_{1}^{*},T_{2}^{*},T_{3}^{*},T_{4}^{*}\right]$. Then, she makes plaintext into ciphertext and transmits them to the recipient Bob. The parameters (*g*,*μ*,*R*,*p*,*q*) are kept confidential. The ciphertext sequences with redundant DNA sequences corresponding to random numbers [*r*_1_,*r*_2_,*r*_3_,*r*_4_] are transmitted over public channels.

(2) Encryption

Firstly, the plaintext is converted into binary strings according to the ASCII code. Then, the encryption key *k* is generated chaotic sequences by substituting the parameters [*g*,*μ*] into the Logistic formula. According to the XOR operation in Section 2, the plaintext binary strings are encrypted with the value of *k* (for example, input *A*' represents "1", input *B*' represents "0"). Thus, the fluorescence intensity spectrum, the ciphertext, is obtained by making use of the well-made self-assembly structure to add the corresponding chains for strand displacement reaction. Finally, the ciphertext is transmitted to the receiver through the security channel.

(3) Reconstruction of the self-assembly structure

The recipient Bob obtains [*r*_1_,*r*_2_,*r*_3_,*r*_4_] according to the random numbers extraction algorithm proposed above, and the self-assembly structure can be reconstructed with other information as described in Section 3.

(4) Decryption

Similar to the encryption, the ciphertext is calculated with the key *k* by adding the corresponding strands to the self-assembly structure. The final result of the fluorescence intensity spectrum is the plaintext.

The detailed procedures of the encryption and decryption algorithms are explained in the following pseudocodes (**Algorithms 1 and 2**).

## Algorithm 1. The encryption algorithm based on the DNA self-assembly structure operation

**Input:** An 8-bit plaintext *M* and all public parameters

**Output:** The cipher fluorescence *C* and r*

(1) *n*: = the available DNA strands set obtained from DNA database;

(2) *p*: = the selected reference strand from set *n*;

(3) [*r*_1_,*r*_2_,*r*_3_,*r*_4_]: = the four different pseudo-random numbers in the interval [1,*L*−16];

(4) [*x*,*y*,*z*,*u*]: = the four binary sequences converted by [*r*_1_,*r*_2_,*r*_3_,*r*_4_];

(5) [*s*_1_,*s*_2_,*s*_3_,*s*_4_]: = the four corresponding DNA sequences replaced with [*x*,*y*,*z*,*u*] according to the mapping of *R*([*A*,*T*,*C*,*G*] and [00,01,10,11]);

(6) [*q*,*s*_1_,*s*_2_,*s*_3_,*s*_4_,*q*',*s*_5_]: = connecting *q* to [*s*_1_,*s*_2_,*s*_3_,*s*_4_] and adding the redundant sequences *s*_5_;

(7) r*: = transmitting [*q*,*s*_1_,*s*_2_,*s*_3_,*s*_4_,*q*',*s*_5_] through the public channel;

(8) [*m*_1_,*m*_2_,*m*_3_,*m*_4_]: = four different DNA segments are intercepted from *p* followed by the starting point [*r*_1_,*r*_2_,*r*_3_,*r*_4_];

(9) [Pyramid Structure]: = constructing the self-assembly structure using all public parameters and [*m*_1_,*m*_2_,*m*_3_,*m*_4_];

(10) *k*: = generating chaotic sequences with substituting [*g*,*μ*] into the Logistic formula, and the length of chaotic sequences is equal to *M*;

(11) [*A*',*B*']: = denoting input strand *A*' and input strand *B*' as binary "1" and "0", respectively;

(12) *C*: = performing the combination operation with *M* and *k* according to the XOR logical gate model;

(13) [*C*,r*]: = obtaining the cipher fluorescence *C* and r*.

## Algorithm 2. The decryption algorithm based on the DNA self-assembly structure operation

**Input:** The cipher fluorescence *C*, r*, [*g*,*μ*], [*R*,*p*,*q*] and all of the public parameters

**Output:** The 8-bit plaintext *M*

(1) [*R*,*q*]: = the coding rules and primer information obtained from [*R*,*p*,*q*]

(2) r*: = the selected strand obtained from the medium by using primer *q*;

(3) [*s*_1_,*s*_2_,*s*_3_,*s*_4_]: = the four corresponding DNA sequences of r* by removing *s*_5_ and *q*;

(4) [*x*,*y*,*z*,*u*]: = the four binary sequences converted to [*s*_1_,*s*_2_,*s*_3_,*s*_4_] according to *R*;

(5) [*r*_1_,*r*_2_,*r*_3_,*r*_4_]: = the four different pseudo-random numbers converted to [*x*,*y*,*z*,*u*];

(6) [*m*_1_,*m*_2_,*m*_3_,*m*_4_]: = the four different DNA segments intercepted from *p* followed by the starting point [*r*_1_,*r*_2_,*r*_3_,*r*_4_];

(7) [Pyramid Structure]: = constructing the self-assembly structure by using all of the public parameters and [*m*_1_,*m*_2_,*m*_3_,*m*_4_];

(8) *k*: = generating chaotic sequences with substituting [*g*,*μ*] into the Logistic formula, and the length of chaotic sequences is equal to *c*';

(9) [*A*″,*B*″]: = denoting input stand *A*″ and input strand *B*″ as binary "1" and "0";

(10) *M*: = performing the combination operation with *C* and *k* according to the XOR logical gate model.

## Enhanced double-bit one-time-pad encryption model

### Advanced calculator model

The single-bit encryption mentioned above uses one strand to represent one binary number of the plaintext and key. To improve the computational efficiency, we propose a double-bit encryption scheme that uses one strand to represent two binary numbers, for example, [*A*',*B*',*C*',*D*'] denote [00,01,10,11], respectively. Obviously, according to the strand displacement described in Section 2, the *RS*_1_-*RS*_2_ joint will not be exposed and no significant fluorescence signal will appear if the same or relative strands are added. However, the output results have two options: 00 or 10. We cannot distinguish the input strand through the fluorescence signal. Similarly, *RS*_1_-*RS*_2_ will be fully exposed and significant fluorescence signal will be noted if two adjacent different strands are added. The output corresponds to two results: 01 and 11. We still can’t distinguish between these results.

**Fig 4:**
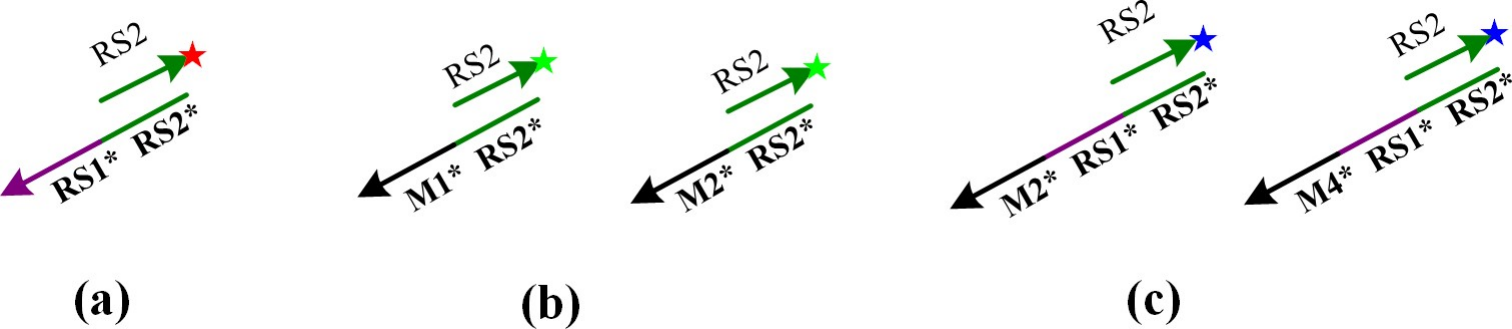
Three different fluorescent color detectors.

Thus, based on the above problems, we use different colors of "detector" to distinguish the different ciphertext results. As shown in Fig 4, we designed three different color detectors: red, green and blue. Here, detector (a) and (c) are used to identify ciphertext results "01" and "11", respectively, when two adjacent strands are added, whereas detector (b) is used to identify ciphertext result "10" when two relative strands are added. Detector is not added for the remainder of the scenario. Thus, by default, no fluorescent signal is detected, and the corresponding cipher result is "00".

### Strand displacement reaction

Assuming that the ssDNA [*A*',*B*',*C*',*D*'] represent [00,01,10,11], respectively. The color of (a), (b), and (c) corresponds the operation results "01, 10, 11", respectively, and the achromatic color represents the result "00".

The reaction diagram of different inputs and results are as follows.

**Fig 5:**
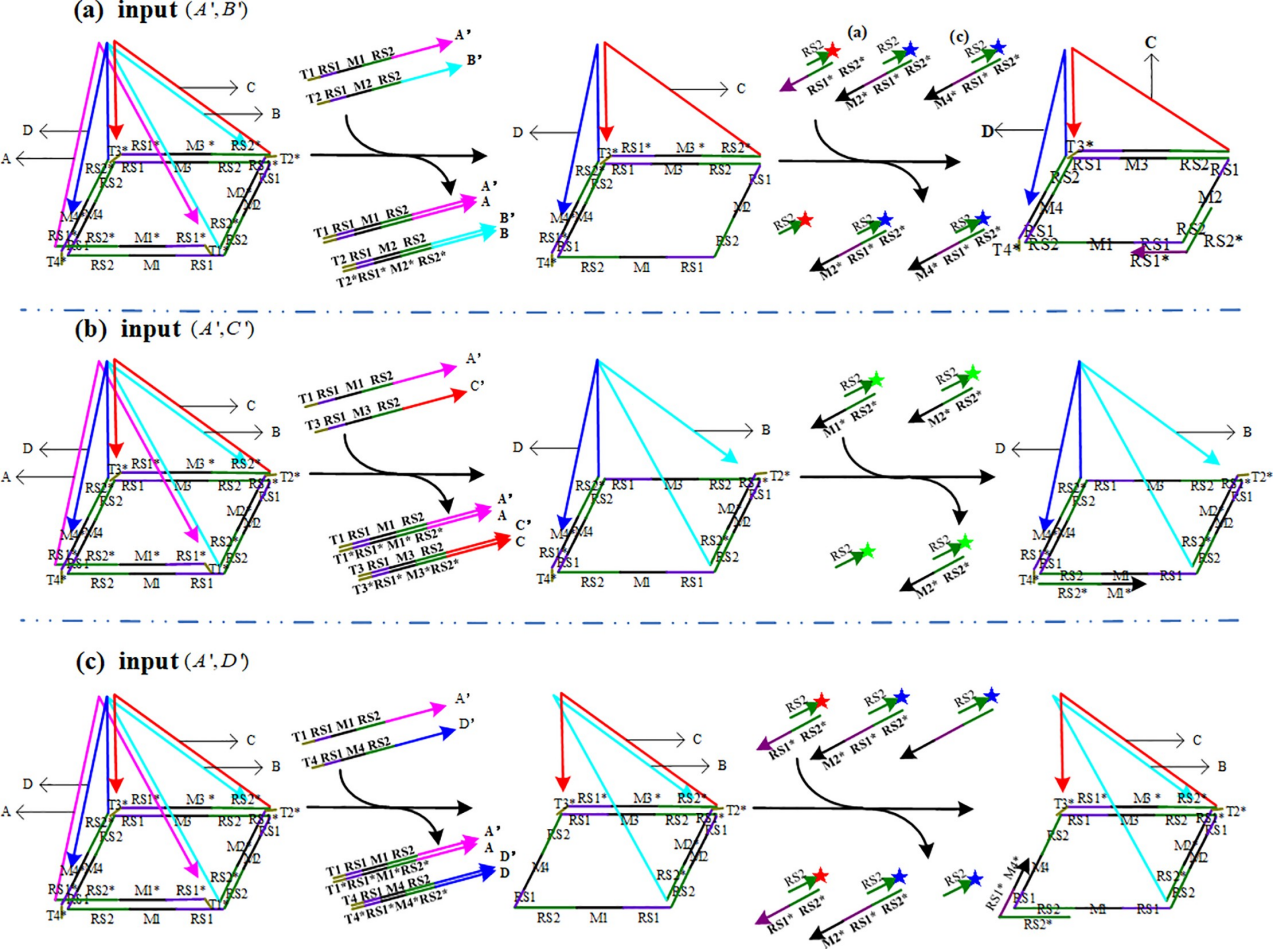
There different displacement processes. (a) The displacement process when adding *A*' and *B*'. (b) The displacement process when adding *A*' and *C*'. (c) The displacement process when adding *A*' and *D*'.

If strands *A*' and *B*'are added (00⊕01 = 01), then detector (a) and (c) are added into the medium. After strand replacement the self-assembly structure will only hybridize with (a), so the result of the fluorescent color is red, as shown in Fig 5(A). The addition of strands *C*' and *D*' will produce the same result.

If strands *A*' and *C*' are added (00⊕10 = 10), detector (b) is added to the medium, and the result of the fluorescent color is green, as shown in Fig 5(B). The addition of (*B*',*D*') produces the same result.

If strands *A*' and *D*' are added (00⊕11 = 11), detector (a) and (c) are added to the medium. Then, the self-assembly structure will only hybridize with (c). Thus, the fluorescent color is blue, as shown in Fig 5(C). Adding strands *C*' and *B*' will produce the same result.

If two of the same strands are added, we do not add any detector. Thus, no fluorescence signal is obtained, corresponding to the result "00". The truth table is presented in **Table 3**.

**Table 3:** Truth table of double-bit operation.

Input		Output
*X*	*Y*	
00	00	00
	01	01
	10	10
	11	11
01	00	01
	01	00
	10	11
	11	10
10	00	10
	01	11
	10	00
	11	01
11	00	11
	01	10
	10	01
	11	00

## Discussion and analysis

### Simple example

Here, we choose the word "*dna*" as plaintext to implement the encryption and decryption operation. According to the ASCII code, "*dna*" is converted into binary strands as follows.

$${\left[''dna''\right]}_{Ascii}=01100100\;01101110\;01100001$$

The encryption key is obtained as follows by substituting [*g* = 0.501,*μ* = 3.68] into the Logistic formula.

$${\left[k\right]}_{Bin}=10111111\;11110101\;11010111$$

(1) Single-bit encryption

The strands *A*' and *B*' represent input "1" and "0", respectively. The production of fluorescent signal is indicated as "1", whereas "0" indicates no signal. The first word is "0", and the corresponding key is "1". When DNA strands *B*' and *A*' are simultaneously added to the medium containing the DNA self-assembly structure, obvious fluorescent signal is observed. Thus, the output is "1". The third word is "1", and the corresponding key is "1". When two strands *A*' are added to the medium, no obvious fluorescence signal is observed. Thus, the output is "0". Given that the XOR operation at each bit is performed individually, all bits of the plaintext are implemented in parallel. Finally, based on fluorescent signals of all bits, we obtained the ciphertext, namely a new binary strand "11011011 10011011 10110110", and sent the fluorescent signals to the receiver in a public channel without worrying about the loss of secret.

(2) Single-bit decryption

The process of decryption is the same as the encryption, and the results are also obtained by detecting fluorescent signals. For the first bit, both of the ciphertext and key are "1". After adding strand *A*' twice to the medium, there is no obvious fluorescence signal. Thus, the first plaintext binary is "0". For the second bit, the ciphertext and key is "0" and "1", respectively. When adding strands *B*' and *A*' to the medium, a clear fluorescence signal appears. Here, the plaintext is "1". Thus, the fluorescent signal of all the bits, that is the plain string, is "01100100 01101110 01100001". According to the coding rules, the binary string can be converted into the plaintext "*dna*".

(3) Double-bit encryption

For the first group, the plaintext and key are "01" and "10", respectively. When strands *B*' and *C*' are added to the medium and detectors (a) and (c) are added, the self-assembly structure will only hybridize with (c). Thus, a clear blue fluorescent signal is evident, and the result is "11". The plaintext and key of the second group are "10" and "11", respectively. When strands *C*' and *D*' are simultaneously added to the medium followed by the detector (a) and (c), there is a clear red fluorescent signal. The corresponding ciphertext is "01". The plaintext and key of the third group are "01" and "11", respectively. Strands *B*' and *D*' are added to fully displace strands *B* and *D*. A green fluorescent signal is generated after adding the detectors (b). Thus, the result is "10". The entire obtained fluorescence signal spectrum is the ciphertext.

(4) Double-bit decryption

The receiver performs the same encryption process to complete the decryption operation according to the ciphertext fluorescence signal spectrum and key. For the first group, the ciphertext is "11", and the corresponding key is "10". Chain *D*' and *C*' are added to the medium. Then, detectors (a) and (c) are added, and a clear red fluorescence signal is obtained. Thus, the first group of plaintext is "01". The second group of the ciphertext and key are "01" and "11", respectively. First, strands *B*' and *D*' are added to the medium. Then, detector (b) is added, and a clear green fluorescence signal is produced. Thus, the second group of plaintext "10" is obtained. Finally, all the fluorescence signal spectrums are obtained. Then, the receiver can convert the fluorescence signal to the corresponding binary strings to obtain the plaintext according to the coding rules.

**Fig 6:**
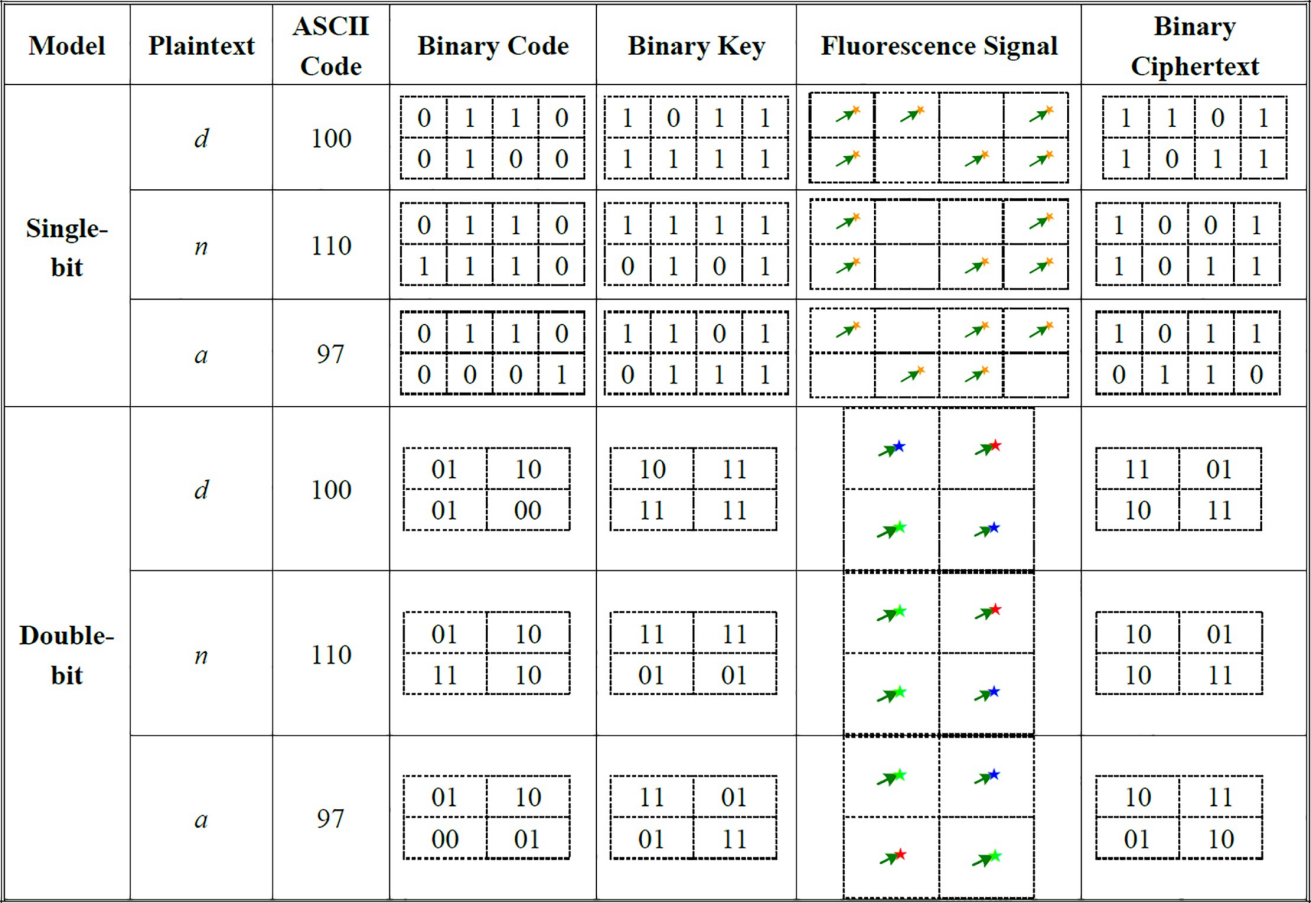
The process of encryption by using two models.

A detailed procedure of the encryption and decryption by using two models are explained in the following Fig 6.

### Secret key’s sensitivity analysis

The encryption key *k* is generated by using the logistic map. Its mathematical define is as follows:

$$x_{n+1}=\mu x_{n}(1-x_{n})$$ (1)

**Fig 7:**
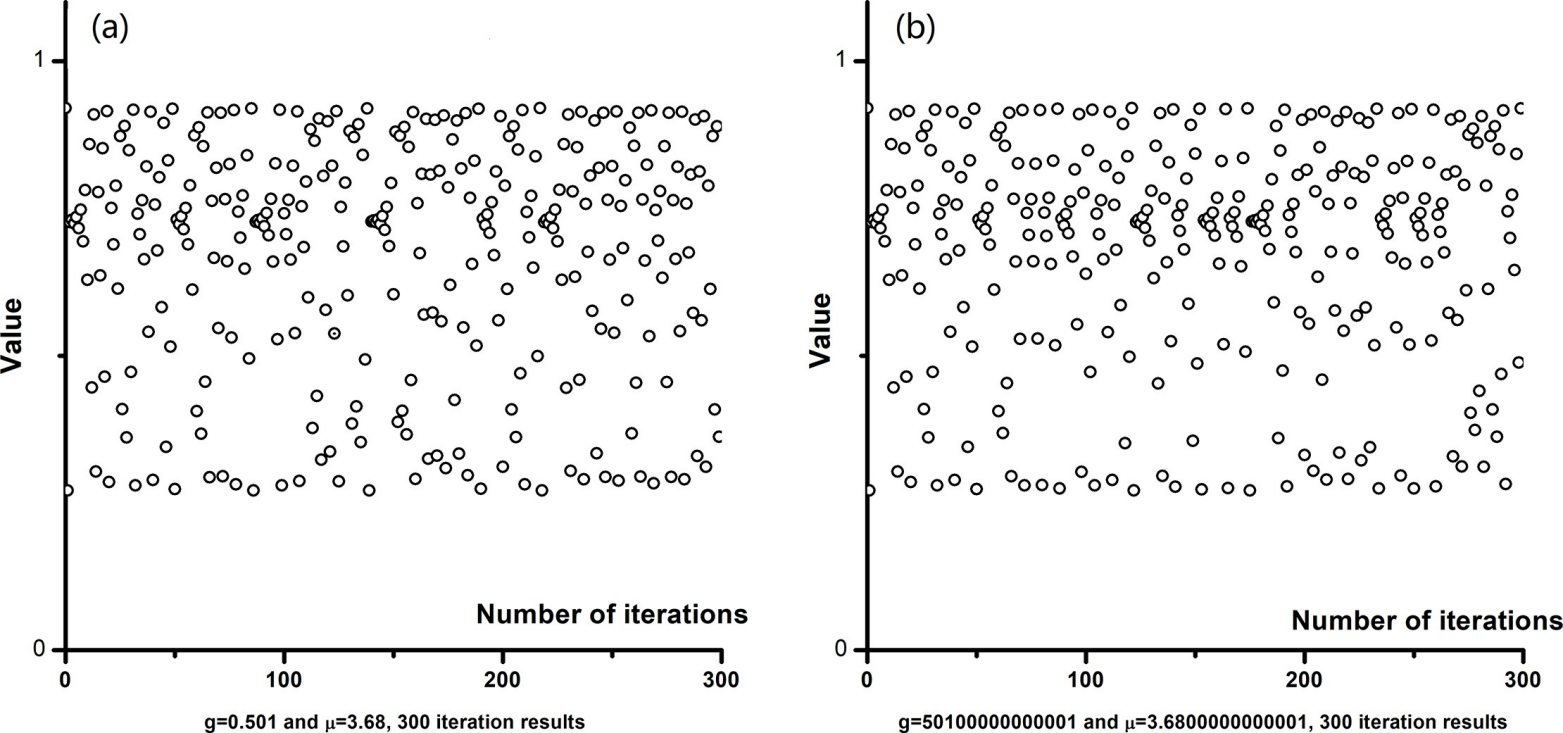
Scatter diagram with 300 iterations: (a) is the iteration value displayed on the 300 times with *g* = 0.501,*μ* = 3.68; (b) shows the iteration value displayed 300 times with *g* = 0.50100000000001,*μ* = 3.6800000000001.

Chaotic sequences have rapid diffusibility and high sensitivity to initial value. In implementation of the algorithm, a slight change of arbitrary parameter in secret key will affect the results of encryption and decryption. In order to test the sensitivity of the secret key, Fig 7 shows the scatter diagram with 300 iterations where

(g=0.501,μ=3.68)

and

(g=0.50100000000001,μ=3.6800000000001).

### Computational security analysis

The traditional cryptography treats the confidentiality and length of the key as the only measure of encryption strength. Once the key is leaked, the security system is broken. Therefore, the cryptography scheme proposed in this paper provides two layers of security by involving biological and computational difficulties.

The calculated security of this scheme is mainly based on the following factors: randomness of the DNA reference strand *p*, randomness of the segments *M*_*i*_(*i* = 1,2,3,4), security of the encrypted transmission of the random numbers *r*_*i*_(*i* = 1,2,3,4) based on the primer information, and randomness of the corresponding selection between input strands [*A*',*B*',*C*',*D*'] and binary bit.

We assume that the number of DNA strand in the DNA database is *N*_1_, the length of the selected target strand and the plaintext is *n* and *n*_1_ respectively, the selection type of the fragments is *N*_2_, the types of the corresponding relationship between the binary coding and the bases [*A*,*T*,*C*,*G*] is *N*_3_, the types of corresponding relationship between the input strand and the binary bit is *N*_4_, the selection types of primer information is *N*_5_ and the length is *n*_2_, and the types of the corresponding relationship between the fluorescence information and the binary bit is *N*_6_. So,

$$P_{1}=\frac{1}{N_{1}{}^{n_{1}}}$$ (2)

$$P_{2}=\frac{1}{N_{2}}=\frac{1}{C_{n-15}^{4}}$$ (3)

$$P_{3}=\frac{1}{N_{3}}=\frac{1}{4!}$$ (4)

$$P_{4}=\frac{1}{N_{4}}$$ (5)

$$P_{5}=\frac{1}{N_{5}}=\frac{1}{C_{4^{n_{2}}}^{2}}$$ (6)

$$P_{6}=\frac{1}{N_{6}}$$ (7)

- *P*_1_: Selection probability of DNA reference strand
- *P*_2_: Selection probability of *M*_*i*_.
- *P*_3_: Selection probability of the corresponding relationship between the binary bit and the base.
- *P*_4_: Selection probability of corresponding relationship between the input strand and the binary bit.
- *P*_5_: Selection probability of the selection type of primer information.
- *P*_6_: Selection probability of the corresponding relationship between the fluorescence information and the binary bit.

The total ciphertext crack probability is

$$\begin{array}{l} P=P_{1}\times P_{2}\times P_{3}\times P_{4}\times P_{5}\times P_{6}\\ \;=\frac{1}{N_{1}{}^{n_{1}}}\times \frac{1}{C_{n-15}^{4}}\times \frac{1}{4!}\times \frac{1}{N_{4}}\times \frac{1}{C_{4^{n_{2}}}^{2}}\times \frac{1}{N_{6}} \end{array}$$ (8)

(1) Single-bit scheme ciphertext crack probability

The ciphertext fluorescence signal spectrum is transmitted publicly. When the attacker obtains the ciphertext signal, it is necessary to judge the corresponding relationship between 0, 1 and the fluorescence intensity (*N*_4_ = 2) and select the strands corresponding 0 and 1 from ssDNA strands (*A*',*B*',*C*',*D*')(*N*_6_ = 4×3 = 12). So,

$$P_{4}=\frac{1}{N_{4}}=\frac{1}{12}$$ (9)

$$P_{6}=\frac{1}{N_{6}}=\frac{1}{2}$$ (10)

In summary, the probability of the final interpretation of the plaintext in the single-bit encryption is:

$$\begin{array}{l} P=P_{1}\times P_{2}\times P_{3}\times P_{4}\times P_{5}\times P_{6}=\frac{1}{N_{1}{}^{n_{1}}}\times \frac{1}{C_{n-15}^{4}}\times \frac{1}{4!}\times \frac{1}{12}\times \frac{1}{C_{4^{n_{2}}}^{2}}\times \frac{1}{2}\\ \;=\frac{1}{N_{1}{}^{n_{1}}\times C_{n-15}^{4}\times C_{4^{n_{2}}}^{2}\times 24^{2}} \end{array}$$ (11)

(2) Double-bit scheme ciphertext crack probability

Firstly, we should take into account the corresponding relationship between (*A*,*B*,*C*,*D*) and (00,01,10,11), namely *N*_4_ = 4! = 24. We should also consider that the corresponding relationship between the four colors (red, green, blue, colorless) and the results (00,01,10,11) is *N*_6_ = 4! = 24. Thus,

$$P_{4}=\frac{1}{N_{4}}=\frac{1}{24}$$ (12)

$$P_{6}=\frac{1}{N_{6}}=\frac{1}{24}$$ (13)

In summary, the probability of the attacker finally obtaining the plaintext in the double-bit encryption is

$$\begin{array}{l} P=P_{1}\times P_{2}\times P_{3}\times P_{4}\times P_{5}\times P_{6}=\frac{1}{N_{1}{}^{n_{1}}}\times \frac{1}{C_{n-15}^{4}}\times \frac{1}{4!}\times \frac{1}{24}\times \frac{1}{C_{4^{n_{2}}}^{2}}\times \frac{1}{24}\\ \;=\frac{1}{N_{1}{}^{n_{1}}\times C_{n-15}^{4}\times C_{4^{n_{2}}}^{2}\times 24^{3}} \end{array}$$ (14)

**Fig 8:**
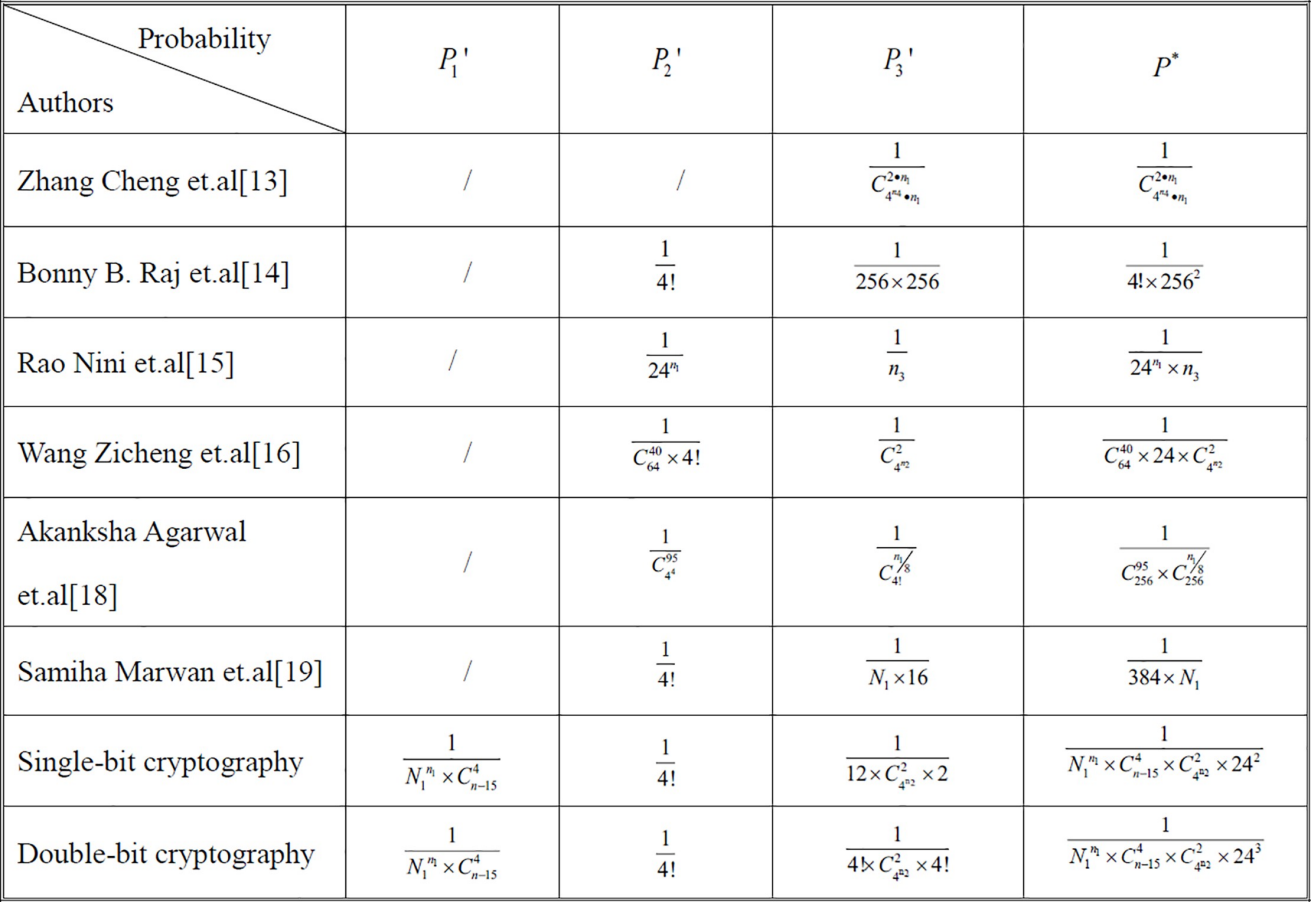
Comparisons of the probability of selection and cracking (here, *n*_1_-the length of plaintext binary; *n*_2_-the length of primer information; *n*_3_-the total number of ring DNA; *n*_4_-the length of the key).

Based on the above calculation of the ciphertext crack probability, a comparison between our schemes and the part of the existing literature is made, as shown in Fig 8. *P*_1_' represents the probability of DNA structure selection, *P*_1_' = *P*_1_×*P*_2_. *P*_2_' represents the probability of coding selection, *P*_2_' = *P*_3_. *P*_3_' represents the probability of ciphertext combination, *P*_3_' = *P*_4_×*P*_5_×*P*_6_. P* represents the total probability of cracking.

**Fig 9:**
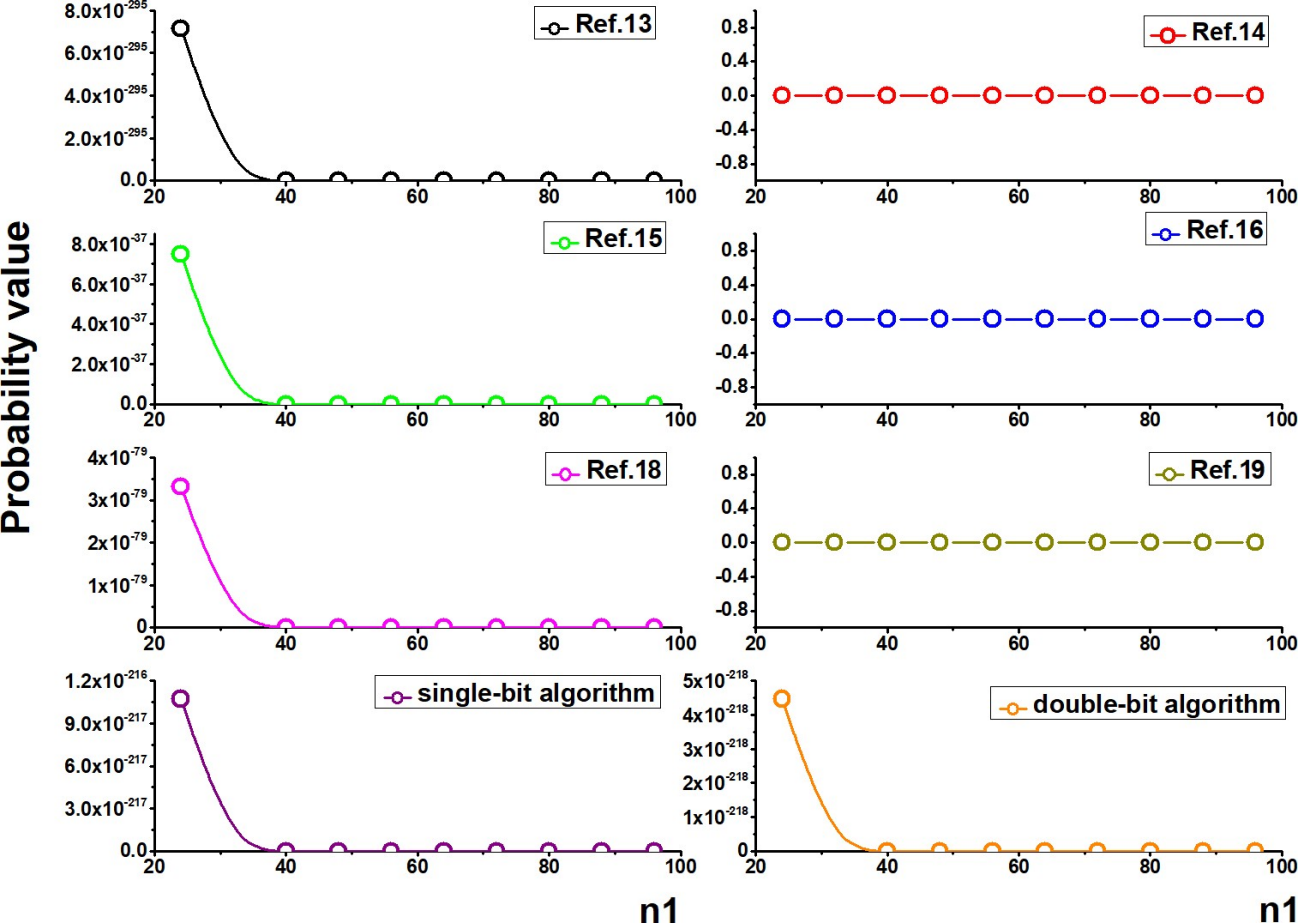
Comparisons of the probability graphs.

There are 1.6×10^8^ real DNA sequences found on the online database (NCBI database), that is, *N*_1_ = 1.6×10^8^. In order to facilitate the probability comparison, we set the length of the reference strand in this paper is 1000*bp*, the length of the primer is 5*bp*, the number of circular DNA in the reference [13] is 1000, the length of the key strand in the reference [16] is 10*bp*, namely *n* = *n*_3_ = 1000,*n*_2_ = 5,*n*_4_ = 10. Therefore, the length of plaintext binary is the only unified variable. The comparisons of probability values and graphs based on the different literatures are as follows **Table 4** and Fig 9.

**Table 4:** Comparisons of the probability values.

*n*_1_	Ref.13	Ref.14	Ref.15	Ref.16	Ref.18	Ref.19	single-bit algorithm	double-bit algorithm
24	7.2E-295	6.4E-07	7.5E-37	3.2E-25	3.3E-79	1.6E-11	1.1E-216	4.5E-218
32	-∞	6.4E-07	6.8E-48	3.2E-25	5.2E-81	1.6E-11	2.5E-282	1.0E-283
40	-∞	6.4E-07	6.2E-59	3.2E-25	1.0E-82	1.6E-11	-∞	-∞
48	-∞	6.4E-07	5.6E-70	3.2E-25	2.5E-84	1.6E-11	-∞	-∞
56	-∞	6.4E-07	5.1E-81	3.2E-25	6.9E-86	1.6E-11	-∞	-∞

According to the above comparisons, it’s obvious that the cracking probability in our schemes and some literatures gradually reduced with the increase in the length of the plaintext while is constant in other literatures. Both of the two algorithms designed in our paper provide higher computational security relative to most existing schemes.

### Resistance attack analysis

Referring to the reference [20], we analyze the security model of the encryption and decryption algorithm proposed in our scheme.

(1) Resists exhaustive key attack

The encryption key is generated by using the Logistic map and the key value depends on the initial parameters. According to the above proposed scheme, the value of parameter *g* ranges from 0 to 1 while the value of parameter *μ* ranges from 3.569945 to 4, so the key space is 0.5×10^32^. Meanwhile, the selection probability of DNA coding mapping rules *R* is 1/24 since it can be selected randomly in the process of plaintext conversion. In addition, there are 24 different mappings between the fluorescence spectrum C and ciphertext code based on the combinations of fluorescence signal with binary bits. According to the formulas (4), (6) and (7), the probability of cracking the true meaning of ciphertext is $1/48\times C_{4^{n_{2}}}^{2}$ in the single-bit scheme while it is $1/24^{2}\times C_{4^{n_{2}}}^{2}$ in the double-bit scheme. Therefore, it provides dual security protection in both computational and biological aspects in our scheme, and it is not feasible to attack ciphertext by using exhaustive key.

(2) Resists self-assembly structure attack

In theory, each plaintext can be encrypted with a distinct self-assembly structure in our scheme. But more importantly, the DNA fragment [*M*_1_,*M*_2_,*M*_3_,*M*_4_] of Pyramid structure is selected from *p* by the random numbers [*r*_1_,*r*_2_,*r*_3_,*r*_4_]. The target strand *p* is randomly selected from NCBI database which contains about 1.6×10^8^ available DNA sequences. Meanwhile, the four different pseudo-random numbers [*r*_1_,*r*_2_,*r*_3_,*r*_4_] take values in the interval [1,*L*−16]. According to the formulas (2) and (3), the probability of cracking self-assembly structure is $1/{\left(1.6\times 10^{8}\right)}^{n_{1}}\times C_{n-15}^{4}$. So, the crack probability gradually decreases with the increasing of plaintext length.

(3) Resists exhaustive plaintext attack

Implementing an exhaustive plaintext attack, the attacker needs to obtain (*g*,*μ*) and *R* so as to get the encryption key and mapping rule firstly, but the cracking probability is 1/48×10^32^. Secondly, the attacker needs to crack the DNA self-assembly structure and algorithm in order to implement the encryption and decryption. According to the above analysis, the cracking probability of self-assembly structure is $1/{\left(1.6\times 10^{8}\right)}^{n_{1}}\times C_{n-15}^{4}$. Therefore, the algorithm can provide more security and resist exhaustive plaintext attack.

(4) Resists statistical analysis attack

The attacker can crack the ciphertext by analyzing the statistical rules of the plaintext and ciphertext. In this scheme, the probability of cracking the true meaning of ciphertext (*C*,r*) is $1/48\times C_{4^{n_{2}}}^{2}$ in the single-bit scheme while it is $1/24^{2}\times C_{4^{n_{2}}}^{2}$ in the double-bit scheme, it provides more computational security. The primers *q* in each ciphertext r* are completely different, and the order of bases in the DNA sequences is more irregular. So, the attacker can’t analyze the statistical law between plaintext and ciphertext using only the ciphertext information (*C*,r*).

(5) Provide forward and backward security

In this scheme, the self-assembly structure and the key generation parameters (*g*,*μ*) which be used to encrypt each segment of plaintext is different. Even if these parameters leak causes the key to be compromised during the current round of encryption, it will not affect the confidentiality of the previous or subsequent ciphertext.

(6) Resists replay attack

The plaintext is divided into many segments. Different segment is encrypted using different self-structure. Therefore, the random number (*r*_1_,*r*_2_,*r*_3_,*r*_4_), the primers *q* and the key parameter (*g*,*μ*) are different. Even if the attacker replays the ciphertext information r*, the recipient cannot intercept the correct parameters (*s*_1_,*s*_2_,*s*_3_,*s*_4_). So the structure cannot be reconstructed. If the current key is replayed, the attacker can only get the current plaintext segment and cannot crack other plaintext segments.

(7) Resists man-in-the-middle attack

By default, the construction method of self-assembly structure and the encryption and decryption algorithms are known information for both encryption and decryption in our scheme. Even if the attacker intercepts all the key parameters [*R*,*p*,*q*] and (*g*,*μ*) from the secret transmission channel and the parameters $\left[T_{1}^{*},T_{2}^{*},T_{3}^{*},T_{4}^{*}\right]$ and ciphertext (*C*,r*) from the public channel, it is impossible to construct the DNA self-assembly structure accurately without knowing the precise biological experiment conditions. The operation of encryption and decryption algorithm is also based on the biological hybrid experiment. Therefore, it is not feasible to spoof the encryption party or the decryption party even if the attacker obtains all the parameters of a certain encryption.

(8) Resists only ciphertext attack

The ciphertext information (*C*,r*) is transmitted in public channel and easy to be intercepted by attacker. In the case of only knowing (*C*,r*), if the attacker wants to crack the plaintext, it also needs to obtain the meaning of ciphertext, the key *k*, the self-assembly structure and the experimental conditions of the biological reaction. Therefore, it is difficult for the attacker to crack and obtain the plaintext information only with the ciphertext based on the computational and biological security provided by the scheme.

(9) Resists known plaintext attack

Suppose the attacker intercepts some pairs of the plaintext *M* and the corresponding ciphertext *C*, the purpose is to obtain the key *k*. The different DNA self-assembly structures can be designed for each plaintext and the key *k* can be generated by using different Logistic initial parameters (*g*,*μ*) in our scheme. Even if the attacker can obtain the DNA self-assembly structure and the *k* for a certain plaintext, it cannot be used for the next time. Moreover, the plaintext is divided into many segments. Different segment is encrypted using different self-structure. The attacker needs to know the segmentation rules and get each pair between plaintext and the ciphertext so as to crack each key segment. Obviously, the key obtained through the above steps is meaningless, and cannot be used for the next decryption.

(10) Resists chosen plaintext attack

The ciphertext information (*C*,r*) is transmitted in the public channel in our scheme. Suppose the attacker wants to obtain the algorithm operations by using the obtained pairs of partial plaintext and the corresponding ciphertext, it needs to compare the acquired ciphertext information and the ciphertext information (*C*,r*) firstly. Therefore, the attacker needs to crack the meaning of the ciphertext information (*C*,r*). The above analysis shows that the cracking probability is $1/48\times C_{4^{n_{2}}}^{2}$ in the single-bit scheme and $1/24^{2}\times C_{4^{n_{2}}}^{2}$ in the double-bit scheme. Following the increase of the parameter *n*_2_, the cracking probability gradually decreases. Even if the attacker succeeded to crack the key and algorithm rules for this time, it is difficult to crack the corresponding plaintext information for the next time without the key *k*. Therefore, the one-time-pad feature of the scheme in this paper can effectively resist chosen plaintext attack.

(11) Resists chosen ciphertext attack

Suppose the self-assembly structure, ciphertext and corresponding plaintext in a certain time can be obtained, the attacker wants to use these pieces of information to obtain all the plaintext information. Perhaps the attacker can crack the key used in this part by using part of the ciphertext and the corresponding plaintext information. However, the plaintext is encrypted in segments in out scheme, both of the self-assembly structures and key generation parameters for each segment are different. Even if the attacker obtains the key used to encrypt the plaintext of any one segment, the obtained key and self-assembly structure cannot be used to decrypt other parts of the plaintext.

### Algorithm complexity analysis

Assume that the time needed for making self-assembled structures is *T*1, the encryption time of the random numbers is *T*2, and the single plaintext encryption time is *T*3. The number of plaintext is *n*_1_, so the total encryption time is *T* = *T*1+*T*2+*T*3×*n*_1_, the corresponding time complexity is *O*(*m*+*n*_1_). If we use different structures to encrypt each plaintext, the total encryption time is $T={\left(T1+T2+T3\right)}^{n_{1}}$, the corresponding time complexity is $O(m^{n_{1}})$. In summary, the algorithm's spatial complexity and computation speed are *O*(*n*_1_) and *n*_1_/*T* respectively.

### Performance analysis

To qualitatively compare the performance of the scheme, the following parameters are defined:

**Definition 2** DNA coding integrity

The DNA coding table should provide DNA encoding sequences for the complete character set. The complete code table should provide alphabets (uppercase, lowercase), numbers and special characters. It is used to encode all the character set of the plaintext into the DNA sequences.

**Definition 3** Dynamic coding table

The Encoding Table is changed randomly after every session interval and providing different DNA sequences for every element of the character set. To ensure an increased level of security, the encoding table should be changed at periodic intervals or for every interaction session between the sender and receiver.

**Definition 4** Structure randomness

In order to improve the security, the DNA calculator structure should be generated randomly according to the selected random numbers every time. That is, the encryption structure should be unique and different every time.

**Definition 5** Biological process simulation

The biological process of all proposed DNA encryption and decryption algorithms should be simulated to adapt to the digital computing environment. Actually, among the existing studies some proposed cryptographic algorithms are not suitable to be applied in the digital computing environment and only purely based on biology laboratory experiment while the other algorithms are based on a biological process simulation and modern cryptography. Given that modern cryptographic algorithms have been broken by DNA cryptography, a complete algorithm, which is based on the simulation of difficult biological processes, is required.

**Definition 6** Results predictability

The results of the experimental process can be accurately predicted, so the problem can be corrected in time.

The detailed comparisons of existing studies are explained in the following **Table 5**.

**Table 5:** Comparisons of existing studies.

Literatures	DNA coding integrity	Dynamic coding table	Structure randomness	Biological process simulation	Results predictability
**Ref.[13]**	✓	✗	✗	✓	✓
**Ref.[14]**	✓	✓	✗	✗	✗
**Ref.[15]**	✓	✗	✗	✓	✗
**Ref.[16]**	✓	✗	✗	✓	✗
**Ref.[18]**	✓	✗	✗	✗	✗
**Ref.[19]**	✓	✓	✗	✗	✗
**Ref.[21]**	✗	✗	✗	✗	✗
**Ref.[22]**	✗	✗	✗	*	✗
**Ref.[23]**	✗	✗	✓	✓	✓
**Ref.[24]**	✗	✗	✗	✓	✗
**Single-bit** **cryptography**	✓	✓	✓	✓	✓
**Double-bit** **cryptography**	✓	✓	✓	✓	✓

✗- Indication of minimum level of support.

✓- Indication of acceptable level of support.

*—Partial fulfillment.

Our scheme meets all of the above-mentioned performance quality parameters. Not only the text information but also the digital information can be transformed into binary strings so as to transform into corresponding DNA sequences. Each session can randomly select a conversion principle from 8 available code combinations. Four core fragments are interrupted from a certain DNA strand selected from the DNA database randomly by random numbers, so the structure is different in encryption and decryption operation every time. In addition, the encryption and decryption operation in this scheme are achieved base on the process of biological hybridization, and the experimental results can be accurately predicted with the addition of known strands.

### Biosecurity analysis

Firstly, all key parameters are hidden in the DNA strand by DNA steganography and sent to recipients. Only by obtaining correct primers can we find effective ciphertext strands in multiple redundant sequences. Secondly, the biological environment of self-assembly structure and small differences in temperature also affect the assembly of the structure. Thirdly, because of the use of biological hybridization in the process of encryption and decryption, the problems encountered in the hybridization process, such as the concentration of the DNA strand in the experiment, are to be considered. Lastly, the mapping rule of the ciphertext fluorescence structure should be considered. Therefore, even if all the keys are obtained by an attacker, the attacker still cannot obtain a fully accurate fluorescence signal without a specific self-assembly structure and strand replacement conditions.

## Conclusion

A three-dimensional DNA self-assembly pyramid structure, as a calculator, is designed to construct two one-time-pad encryption algorithms in our scheme. By programming DNA interactions, the calculator has achieved four common logical operations (AND, OR, NOT, XOR). All kinds of plaintext information can be transformed into a corresponding DNA sequence by randomly selecting a conversion principle from 8 available code combinations. The calculator structure is different in encryption and decryption operation every time. Even if the DNA self-assembly structure of a certain encryption is revealed, the difficulty of cracking the other self-assembly structure is not reduced. In addition, the encryption and decryption operation in this scheme are achieved based on the process of biological hybridization, and the experimental results can be accurately predicted with the addition of known strands. The cracking probability is gradually reduced with the increase in the length of the plaintext. Both of the single-bit and double-bit one-time-pad algorithms provide higher computational security.

This paper mainly describes the encryption and decryption scheme using DNA self-assembly structure. As above mentioned, the proposed scheme can also be applied to the authentication and application in the telemedicine information system. Compared with other authentication technologies, the 4FA authentication scheme is more innovative by using the DNA self-assembly technology, and provides dual security in both computational and biological. This will also be the main direction of our next application research. Although our design and schemes are still looking for more theory than practicality, it still provides an alternative strategy to construct complex DNA cryptography. Future research is mainly carried out in four aspects. These aspects include extending the calculator model into four-input majority gate, designing biochemical reactions to perform complex computations, and designing two-dimension Logistic mapping, especially in conjunction with the research work of authentication scheme.

## References

[1] Liang C, Yang J, Zhang C. Research Progress for DNA Cryptography. Net info Security. 2015; 1: 66–71. 10.3969/j.issn.1671-1122.2015.01.012

[2] Adleman L. Molecular computation of solutions to combinatorial problems. Science 1994; 266: 1021–1023. 10.1126/science.7973651 7973651

[3] Yang J, Zhang C, Xu J, Liu XR, Qiang XL. A novel computing model of the maximum clique problem based on circular DNA. Sci China Inf Sci. 2010; 53: 1409–1416. 10.1007/s11432-010-4009-6

[4] Xiao G, Lu M, Qin L, Lai X. New field of cryptography: DNA cryptography. Chin Sci Bull. 2006; 51: 1413–1420. 10.1007/s11434-006-2012-5

[5] Zhang C, Yang J, Xu J. Molecular logic computing model based on self-assembly of DNA nanoparticles. Chin Sci Bull 2011; 56: 3566–3571. 10.1007/s11434-011-4725-3

[6] Li W, Yang Y, Yan H, Liu Y. Three-Input Majority Logic Gate and Multiple Input Logic Circuit Based on DNA Strand Displacement. Nano Lett. 2013; 13: 2980–2988. 10.1021/nl4016107 23710909

[7] Zhu J, Zhang L, Dong S, Wang E. Four-Way Junction-Driven DNA Strand Displacement and Its Application in Building Majority Logic Circuit. ACS Nano. 2013; 7(11): 10211–10217. 10.1021/nn4044854 24134127

[8] Zhu J, Zhang L, Li T, Dong S, Wang E. Enzyme-free unlabeled DNA logic circuits based on toehold-mediated strand displacement and split G-quadruplex enhanced fluorescence. Adv Mater. 2013; 25(17): 2440–2444. 10.1002/adma.201205360 23447454

[9] Zhang C, Yang J, Xu J. Circular DNA logic gates with strand displacement. Langmuir the Acs Journal of Surfaces & Colloids. 2010; 26(3): 1416–9. 10.1021/la903137f 19957974

[10] Sarkar, Mayukh, Ghosal P. Post CMOS Computing Beyond Si: DNA Computer as Future Alternative. IEEE International Conference on Nanoelectronic and Information Systems IEEE. 2016; 10.1109/iNIS.2016.039

[11] Lu MX, Lai XJ, Xiao GZ, Qin L. Symmetric-key cryptosystem with DNA technology. Science in China: Information Sciences. 2007; 50(3): 324–333. 10.1007/s11432-007-0025-6

[12] Gehani A, LaBean TH, Reif JH. DNA-based cryptography. Lecture Notes in Computer Science. 2004; 2950: 34–50.

[13] Yang J, Ma J, Liu S, Zhang C. A molecular cryptography model based on structures of DNA self-assembly. Chinese Science Bulletin. 2014; 59(11): 1192–1198. 10.1007/s11434-014-0170-4

[14] Bonny B, Frank J, Mahalakshmi T. Secure Data Transfer through DNA Cryptography using Symmetric Algorithm. International Journal of Computer Applications 2016; 133.

[15] Rao N. A Cryptosystem Based on Recombinant DNA Technicue. Acta Electronica Sinica. 2004; 32(7): 1216.

[16] Wang ZC, Zhao XH, Wang H, Cui GZ. One-time-pad cryptography algorithm based on DNA cryptography. Computer Engineering and Applications. 2014; 50(15): 97–100.

[17] Siddiqui Z, Abdullah AH, Khan MK, Alghamdi A S. Smart environment as a service: three factor cloud based user authentication for telecare medical information system. Journal of Medical Systems. 2014; 38(1):1–14. 10.1007/s10916-013-0001-1 24346931

[18] Agrawal A, Bhopale A, Sharma J, Ali MS, Gautam D. Implementation of DNA algorithm for secure voice communication. International Journal of Scientific & Engineering Research. 2012.

[19] Marwan S, Shawish A, Nagaty K. DNA-Based Cryptographic Methods for Data Hiding in DNA Media. Bio Systems. 2016; 150: 110–118. 10.1016/j.biosystems.2016.08.013 27634362

[20] Kumari S, Khan MK. More secure smart card‐based remote user password authentication scheme with user anonymity. Security & Communication Networks. 2015; 7(11):2039–2053.

[21] Lu MX, Lai XJ, Xiao GZ, Qin L. Symmetric-key cryptosystem with DNA technology. Science in China: Information Sciences. 2007; 50(3): 324–333. 10.1007/s11432-007-0025-6

[22] Sabry M, Hashem M, Nazmy T. Three Reversible Data Encoding Algorithms based on DNA and Amino Acids Structure. International Journal of Computer Applications. 2012; 54: 0975–8887.

[23] Ning K. A Pseudo DNA Cryptography Method. Computer Science. 2009 10.1016/j.compeleceng.2012.02.007

[24] Sadeg S, Gougache M, Mansouri N, Drias H. An encryption algorithm inspired from DNA. International Conference on Machine and Web Intelligence. IEEE. 2010; 349: 344–349. 10.1109/ICMWI.2010.5648076

